# Novel modes in a Wilson-Cowan network

**DOI:** 10.1186/1471-2202-16-S1-P271

**Published:** 2015-12-18

**Authors:** Jeremy Neuman, Jack D Cowan, Wim van Drongelen

**Affiliations:** 1 Dept. of Physics, University of Chicago, Chicago, IL 60637, USA; 2 Dept. of Mathematics, University of Chicago, Chicago, IL 60637, USA; 3 Dept. of Pediatrics, University of Chicago, Chicago, IL 60637, USA

## 

Spontaneous [1] and synaptically-driven neural activity exhibit a wide variety of dynamics. In the latter case, recent experiments using spike-triggered LFPs [2] have been able to classify stimulated behavior into two distinct categories: 1) traveling waves with smooth attenuation when the input is weak; and, 2) localized responses when the impulse is strong. Unfortunately, our knowledge of the mechanisms behind these differences is lacking on both the cellular and network scales.

This study, employing the spatiotemporal mean-field Wilson-Cowan equations [3], provides a model for the nature of these two modes at the population level. Just as in [2], we detect damped traveling waves with exponential decay when the input is relatively small. When the stimulus increases, the activity stays localized as evidenced by the large slope in the peaks of the activity.

## Conclusions

Understanding the contrast between traveling and localized activity in synaptically-driven neural networks is an important task due to its wide range of applications to areas such as vision, sleep and epilepsy. Here, it was shown that a simple population model exhibits both modes of behavior. This result allows us to further study how various network properties such as density and connectivity strength give rise to these kinds of activity.

**Figure 1:**
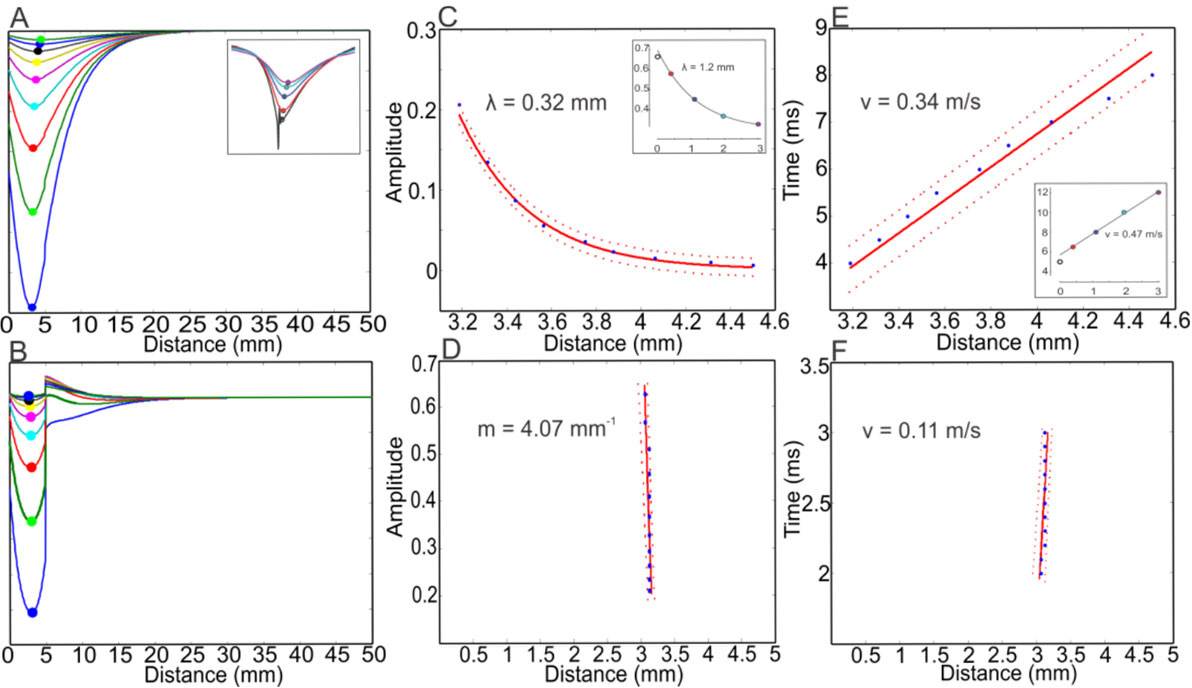
**Simulations showing damped traveling waves and a localized response when the input strength is varied**. (A-B) Diagrams of total activity at equally-spaced times. (C-D) Plots of distance traveled for the peaks (as percentage of global maximum) of curves in (A-B), respectively. (C) is fitted to an exponential as in [2] while (D) fits a linear curve with slope that can be approximated as infinity on the given scale. (E-F) Plots of the rate of propagation of the peaks in (A-B), respectively. Both fits are linear but the localized behavior moves at a much smaller speed. Insets are reproduced from [2].
